# ApoA-I-Mediated Lipoprotein Remodeling Monitored with a Fluorescent Phospholipid

**DOI:** 10.3390/biology8030053

**Published:** 2019-07-12

**Authors:** Edward B. Neufeld, Masaki Sato, Scott M. Gordon, Vinay Durbhakula, Nicolas Francone, Angel Aponte, Gizem Yilmaz, Denis Sviridov, Maureen Sampson, Jingrong Tang, Milton Pryor, Alan T. Remaley

**Affiliations:** 1 Lipoprotein Metabolism Laboratory, Translational Vascular Medicine Branch, National Heart, Lung and Blood Institute, National Institutes of Health, Bethesda, MD 20892, USA; 2 Department of Laboratory Medicine, Clinical Center, National Institutes of Health, Bethesda, MD 20892, USA; 3 Proteomics Core, National Heart, Lung and Blood Institute, National Institutes of Health, Bethesda, MD 20892, USA

**Keywords:** phospholipids/phosphatidylethanolamine, cholesterol/efflux, apolipoproteins, HDL (High-density lipoprotein)/metabolism, lipoproteins, mass spectrometry, membranes/model, low-density lipoprotein (LDL)

## Abstract

We describe simple, sensitive and robust methods to monitor lipoprotein remodeling and cholesterol and apolipoprotein exchange, using fluorescent Lissamine Rhodamine B head-group tagged phosphatidylethanolamine (*PE) as a lipoprotein reference marker. Fluorescent Bodipy cholesterol (*Chol) and *PE directly incorporated into whole plasma lipoproteins in proportion to lipoprotein cholesterol and phospholipid mass, respectively. *Chol, but not *PE, passively exchanged between isolated plasma lipoproteins. Fluorescent apoA-I (*apoA-I) specifically bound to high-density lipoprotein (HDL) and remodeled *PE- and *Chol-labeled synthetic lipoprotein-X multilamellar vesicles (MLV) into a pre-β HDL-like particle containing *PE, *Chol, and *apoA-I. Fluorescent MLV-derived *PE specifically incorporated into plasma HDL, whereas MLV-derived *Chol incorporation into plasma lipoproteins was similar to direct *Chol incorporation, consistent with apoA-I-mediated remodeling of fluorescent MLV to HDL with concomitant exchange of *Chol between lipoproteins. Based on these findings, we developed a model system to study lipid transfer by depositing fluorescent *PE and *Chol-labeled on calcium silicate hydrate crystals, forming dense lipid-coated donor particles that are readily separated from acceptor lipoprotein particles by low-speed centrifugation. Transfer of *PE from donor particles to mouse plasma lipoproteins was shown to be HDL-specific and apoA-I-dependent. Transfer of donor particle *PE and *Chol to HDL in whole human plasma was highly correlated. Taken together, these studies suggest that cell-free *PE efflux monitors apoA-I functionality.

## 1. Introduction

Lipoproteins are composed of a monolayer surface of phospholipids, cholesterol, apolipoproteins and a host of other surface-associated proteins that surround a hydrophobic lipid core of triglycerides and cholesterol ester. The complexity of the protein composition of plasma lipoproteins has been underscored by recent proteomic analyses of very low-density lipoprotein (VLDL), low-density lipoprotein (LDL) and most notably, high-density lipoprotein (HDL) [1,2,3,4]. The composition and organization of lipids on the surface of lipoproteins has also increasingly been the subject of investigation due to their role as substrates and ligands for a number of lipoprotein-modifying enzymes and transfer proteins, as well as for their potential role in regulating the structural organization of functional lipid–protein assemblies on the surface of lipoproteins [5,6].

Complex interactions of plasma enzymes and cofactors with lipoproteins modify both their surface lipid and protein composition and core lipid composition, enabling lipoproteins to serve their various functions in systemic cholesterol and triglyceride energy homeostasis. The dynamic transfer of lipid and exchangeable apolipoprotein (AI, AII, CII, CIII, E) components between lipoproteins has also become an area of increased interest [7,8]. The exchangeability of apoA-I has recently been shown to correlate with its ability to mediate cellular cholesterol efflux [8] and potentially, with cardiovascular disease risk [9]. Recent evidence suggests that perturbed lipoprotein remodeling and lipid and protein exchange may play a causal role in a variety of disorders, including, diabetes [10,11], obesity [12,13], nephropathy [14,15], and cardiovascular disease [9,11].

A variety of methods typically employing radiolabeled lipids and proteins have been used in the past to identify the complex remodeling and component exchange that occurs between lipoproteins. Aside from the reduced cost and safety concerns compared to radiolabeled tags, the use of fluorescent tags has many other advantages, such as ease of detection, simultaneous detection of multiple labels, multi-modal detection capabilities, and the ability to monitor movement of fluorescent tags by microscopy in real time. Fluorescent Bodipy cholesterol has been validated for use as a surrogate for radiolabeled cholesterol in assays that monitor cell-mediated cholesterol efflux [16], cell-free cholesterol efflux [17,18] and Lecithin:Cholesterol Acyltransferase (LCAT) activity [19]. Fluorescent Bodipy cholesterol, like cholesterol, can desorb from lipid surfaces and thus, readily exchanges by passive mechanisms between donor and acceptor lipid surfaces. Similarly, phospholipids labeled with fluorophores covalently attached to an sn-2 short acyl chain can desorb from a lipid surface and readily exchange between lipid surfaces and aqueous acceptors. Their desorption is facilitated by the bulky fluorophore present in the acyl chain, which perturbs the integration of the phospholipid into a lipid monolayer or bilayer. In contrast, head-group labeled phospholipids have acyl chains that are similar to those of endogenous lipids and thus integrate into a lipid surface in a manner similar to endogenous phospholipids. Compared to cholesterol (t½ ~ min,) the rate of intermembrane exchange of phospholipids between lipoproteins is markedly slower (t½ ~ h to days) [20]. Therefore, a head-group-labeled fluorescent phospholipid could potentially serve as a non-exchangeable lipoprotein reference marker in short duration assays (mins to hrs).

Herein, we used head-group-labeled fluorescent phosphatidylethanolamine (PE) as a lipoprotein reference marker, enabling the monitoring of fluorescent cholesterol and apoA-I exchange between lipoproteins, as well as apoA-I-mediated remodeling of lipoproteins. Based on these findings, we developed a new model system to study lipid transfer from donor to acceptor membranes by depositing phosphatidylcholine and cholesterol together with trace amounts of fluorescent lipid-tagged phosphatidylethanolamine (*PE) and cholesterol (*Chol) on calcium silicate hydrate crystals to form dense lipid-coated donor particles that are readily separated from acceptor lipoproteins by centrifugation. We validated the use of this donor–acceptor fluorescent lipid transfer system as a means to study HDL lipoprotein metabolism in vitro by demonstrating that specific transfer of donor particle *PE to plasma HDL is modulated by LCAT and is apoA-I dependent.

## 2. Materials and Methods

### 2.1. Reagents

Phosphatidylcholine, Bodipy cholesterol (23-(dipyrrometheneboron difluoride)-24-norcholesterol; TopFluor Cholesterol), (1,2-dioleoyl-sn-glycero-3-phosphoethanolamine–N-(lissamine rhodamine B sulfonyl)), 1,2-dimyristoyl-sn-glycero-3-phosphocholine (DMPC) and egg lecithin were obtained from Avanti Polar Lipids, Inc. (Alabaster, AL, USA) Lipid Removal Adsorbent (LRA) and cholesterol were obtained from Supelco. All other reagents were obtained from Sigma-Aldrich, unless noted otherwise.

### 2.2. Animals

Plasma was prepared from male mouse blood collected from the periorbital sinus of the eye, as described earlier, and lipids were measured enzymatically, as previously described [21]. All mice including wild-type mice were on a C57BI/6N background. LCAT-knockout (KO) mice [22] (MMRRC Stock No. 11840-MU) were obtained from Jackson Laboratory (JAX), (Bar Harbor, ME, USA) and LCAT Tg mice were generated as described [23]. ApoA-I KO (stock 002055; B6.129P2-Apoa1tm1Unc/J) and ApoA-I Tg mice (stock 1927C57BL/6-Tg(APOA1)1Rub/J) were obtained from JAX. Mice were housed under controlled conditions, with a 12/12 h light/dark cycle, and were fed ad libitum either a standard rodent autoclaved chow diet containing 4.0% fat (NIH31 chow diet; Zeigler Brothers Inc., Gardners, PA, USA). All animal experiments were approved by the Animal Care and Use Committee of the NHLBI (NIH Protocol #H-0050).

### 2.3. Fluorescent Lipid Exchange between Lipoproteins

Pooled and individual human plasma samples were obtained from healthy and dyslipidemic donors. Blood collection was carried out following the rules of the Declaration of Helsinki of 1975 (https://www.wma.net/what-we-do/medical-ethics/declaration-of-helsinki/), revised in 2013. VLDL, LDL, and HDL subfractions were obtained by differential ultracentrifugation [24]. Lipoproteins were labeled with fluorescent PE and cholesterol either alone or together by adding 2 µL of fluorescent lipid stock solution (1 mg/mL ethanol) to 200 µL of human plasma or isolated VLDL, LDL, or, HDL. The reaction mixture was gently mixed and then incubated overnight at 37 °C with mixing (700 rpm) using an Eppendorf 5436 Thermomixer (Eppendorf AG, Hamburg, Germany). Human apoA-I, isolated as previously described [24]), was labeled with Alexa-647 as per the manufacturer’s instructions (Invitrogen, Carlsbad, CA, USA). To monitor cholesterol exchange between lipoproteins, 20 µL of VLDL, LDL, or HDL, isolated from human plasma labeled with both fluorescent PE and cholesterol, were added to 20 µL of unlabeled VLDL, LDL, or HDL, together with 4 µL Alexa-647-tagged human apoA-I (1 mg/mL) and 16 µL of PBS (total reaction mixture of 60 µL) in 1.5 mL plastic conical tubes. Reference standard lipoproteins were prepared by adding 20 µL labeled lipoprotein to 40 µL PBS. Reference standard Alexa-647-tagged human apoA-I contained 4 µL fluorescent apoA-I and 56 µL PBS. Reaction mixtures were gently mixed and then incubated overnight at 37 °C, as above.

### 2.4. Synthetic Lipoprotein X Preparation

Multilamellar lipoprotein-X (LpX) particles containing 24 mole% cholesterol were formed by combining 24.4 mg L-α-lecithin (32 µmoles) together with 4.25 mg cholesterol (10 µmoles) from their respective stock solutions in chloroform. Fluorescent synthetic LpX particles included the addition of 171 µg (130 nmoles) fluorescent-tagged PE (1,2-dioleoyl-sn-glycero-3-phosphoethanolamine–N-(lissamine rhodamine B sulfonyl)), and/or 74 µg (128 nmoles) fluorescent Bodipy cholesterol (23-(dipyrrometheneboron difluoride)-24-norcholesterol) in chloroform. The lipid mixtures were dried under nitrogen. Two milliliters of PBS was added to the dried lipids, and then, the lipids in buffer were vortexed for 10 min to re-suspend the dried lipids. Next, the solution was sonicated for 10 min, using 1 min bursts separated by a 15 s rest interval to generate multilamellar particles. The cholesterol and phospholipid composition of the synthetic LpX particles was confirmed using enzymatic colorimetric assays (Waco, TX, USA) [25].

### 2.5. Lipoprotein X Metabolism In Vitro

Synthetic LpX labeled with fluorescent PE and cholesterol either alone or together (20 µL containing 288 µg of total lipid) were incubated overnight at 37 °C with 20 µL pooled human plasma and sufficient PBS for a total reaction mixture volume of 70 µL. For Fast Protein Liquid Chromatography (FPLC) analysis, synthetic LpX labeled with fluorescent PE and cholesterol (40 µL containing 576 µg of total lipid) was incubated overnight at 37 °C with 20 µL pooled human plasma and 10 µL PBS. In other experiments, unlabeled synthetic LpX, or synthetic LpX labeled with fluorescent PE and cholesterol either alone or together (20 µL containing 288 µg total lipid) was incubated overnight at 37 °C with excess Alexa-647-tagged apoA-I (6 µL containing 6 µg apoA-I) and sufficient PBS for a total reaction mixture volume of 70 µL. Reaction mixtures were gently mixed and then incubated overnight at 37 °C, as above.

### 2.6. rHDL Preparation

Reconstituted HDL (rHDL) was prepared as described previously [26,27], with a final molar ratio of apolipoprotein A-I to soybean phosphatidylcholine of 1:150.

### 2.7. Lipid-Coated LRA Preparation

Synthetic lipids were mixed as described above, using DMPC instead of egg lecithin (DMPC:Cholesterol mole ratio 4:1) and with 5X fluorescent PE and cholesterol to increase the acceptor lipoprotein fluorescent signal to optimal levels. Preliminary studies revealed that substituting lecithin with DMPC improved fluorescent PE transfer to lipoproteins. We used lipid removal absorbent (LRA) as a source of calcium silicate hydrate crystals, with a mean diameter of 10 µM. Lipid-coated particles containing 21 mole % cholesterol were formed by combining 14 mg DMPC (21 µmoles) together with 2.12 mg cholesterol (5.48 µmoles) and the addition of 305 µg (200 nmoles) fluorescent-tagged PE (1,2-dioleoyl-sn-glycero-3-phosphoethanolamine–N-(lissamine rhodamine B sulfonyl)), and/or 27.5 µg (50 nmoles) fluorescent Bodipy-cholesterol (23-(dipyrrometheneboron difluoride)-24-norcholesterol) from their respective stock solutions in chloroform. The lipid mixtures were dried under nitrogen. To form lipid-coated LRA particles, 80 mg of LRA was added along with 2 mL of PBS to the dried lipid mixture and then vortexed for 10 min. The resulting lipid-coated LRA particles were pelleted by centrifugation (1000 rpm, 1 min), and the supernatant was removed and replaced with 2 mL PBS. This washing process was repeated 5 times to ensure removal of any potential lipid vesicles not attached to LRA. For mouse plasma studies, which utilize much smaller plasma volumes than human plasma studies, the LRA particle lipid composition was optimized by increasing the DMPC:Chol ratio to 2:1 in order to enhance fluorescent PE transfer. Lipid-coated particles were formed by combining 11.8 mg DMPC (17.7 µmoles) together with 3.39 mg cholesterol (8.8 µmoles), 305 µg (200 nmoles) fluorescent-tagged PE (1,2-dioleoyl-sn-glycero-3-phosphoethanolamine–N-(lissamine rhodamine B sulfonyl)), and/or 27.5 µg (50 nmoles) fluorescent Bodipy cholesterol (23-(dipyrrometheneboron difluoride)-24-norcholesterol from their respective stock solutions in chloroform, and using 40 mg LRA. The minimum lipid to LRA ratio (mg/mg) of ≈ 4:1 was based on the previously determined lipid binding capacity of LRA [3]. This lipid to LRA ratio provides sufficient lipid to completely cover the surface of LRA particles, thereby preventing direct lipoprotein binding to non-lipid-coated LRA surfaces. We confirmed experimentally that lipoproteins do not bind to lipid-coated LRA particles by showing that *PE-labeled plasma did not transfer *PE to non-fluorescent lipid-coated LRA particles.

### 2.8. In Vitro Studies of Fluorescent Lipid Transfer from Lipid-Coated LRA Particles to Lipoproteins

Pooled or individual human plasma samples (20 µL) were incubated with fluorescent PE- and cholesterol-tagged lipid-coated LRA (60 µL) and PBS (20 µL) for 4 h at 37 °C with mixing. Samples were then centrifuged at 1000 rpm for 1 min to pellet fluorescent lipid LRA particles. Supernatant samples were analyzed by agarose electrophoresis and FPLC. Lipid and lipoprotein composition of the pooled human plasma samples were determined by colorimetric assays for total cholesterol (TC), cholesterol, and triglycerides (TG) (Waco). Based on their composition, samples were designated as: low TG (LTG), high TG (HTG), low LDL (LL), and high HDL (HH). Total Cholesterol: 160.3, 227.5, 120.2, and 201.6, mg/dL, respectively; HDL: 66.9, 39.2, 43.7, and 84.1, mg/dL, respectively; Triglycerides: 37.9, 412.9, 114.7 and 60.9 mg/dL, respectively. Triplicate samples of mouse plasma (10, 15, 20, or 25 µL) were incubated with fluorescent PE and cholesterol-tagged lipid-coated LRA (50 µL) and sufficient PBS for a total volume of 200 µL in 96-well plates for 1 h at 37 °C with mixing (1200 rpm). Following the incubation, an additional 100 µL of PBS was added to each well, and then, the plates were centrifuged at 2000 rpm for 2 min. A total of 10 µL of supernatant from each triplicate sample was pooled for agarose gel electrophoresis analysis.

### 2.9. Fluorescent Lipid Agarose Gel Electrophoresis

Fluorescent lipoproteins were monitored by electrophoresis of 10 µL of reaction mixture or plasma samples, using Sebia Hydragel Lipoprotein(E) 15/30 agarose gels, which ran for 45, 60, or, 90 min as indicated in the Figure Legends. Fluorescent bands on the gel were imaged using a Typhoon 9400 Variable Mode Imager (GE). Fluorescent PE, cholesterol, and apoA-I were detected using excitation/emission wavelengths of 532/560 nm, 488/520 nm, and 633/670 nm, respectively. Following imaging of fluorescent lipids and protein, gels were stained with Sudan Black and rescanned. Quantitative analysis of fluorescent band intensity was performed using ImageQuant 5.1 software.

### 2.10. FPLC Analysis

A quantity of 370 µL of plasma was applied to tandem Superose 6 10/300 GL columns on an AKTA FPLC (Akta Pure, GE Healthcare, Marlborough, MA, USA) and 0.5 mL fractions were collected.

### 2.11. Total PE and Cholesterol Fluorescence Measurements

For in vitro studies, 40 µL of reaction mixture supernatant was diluted with 200 µL of 1% Triton X-100 to solubilize lipids. PE Lissamine Rhodamine B and Bodipy cholesterol fluorescence was measured with a Perkin Elmer Victor3 1420 Multichannel Counter (Perkin Elmer, Waltham, MA, USA) using 540/600 and 485/520 excitation/emission filters, respectively. The fluorescence emission/ng fluorescent lipid for lipid-coated LRA particles was determined by extracting various volumes of fluorescent PE and cholesterol-labeled lipid-coated LRA in TX-100 detergent and measuring Lissamine Rhodamine B and Bodipy fluorescence, as described above. The mass of fluorescent PE and cholesterol per unit volume lipid LRA was then used to calculate fluorescence emission/ng of lipid LRA.

### 2.12. In-Gel Proteomic Analysis

Agarose gel bands representing HDL or the band above HDL (15 lanes) were scraped from the gel plastic backing and transferred to plastic Eppendorf tubes. Samples were subjected to in-gel reduction by dithiothreitol and carbamidomethylation by iodoacetamide, followed by overnight digestion with sequencing-grade trypsin. Protein digests were filtered to separate peptides from gel fragments and desalted using ZipTip C18 columns (Millipore, Burlington, MA, USA). Samples were re-suspended in 0.1% formic acid and analyzed by nanoLC-MS/MS on an Orbitrap Elite mass spectrometer (Thermo Scientific, Waltham, MA, USA). Mass data was searched against the Swiss-Prot database using Mascot search engine and Proteome Discover software (Thermo, Waltham, MA, USA). Identifications were validated using Scaffold (Proteome Software) with both peptide and protein thresholds set to 95% confidence and a minimum of 2 peptides for protein identification.

## 3. Results

### 3.1. Incorporation of Fluorescent PE and Cholesterol into Lipoproteins

**Figure 1:**
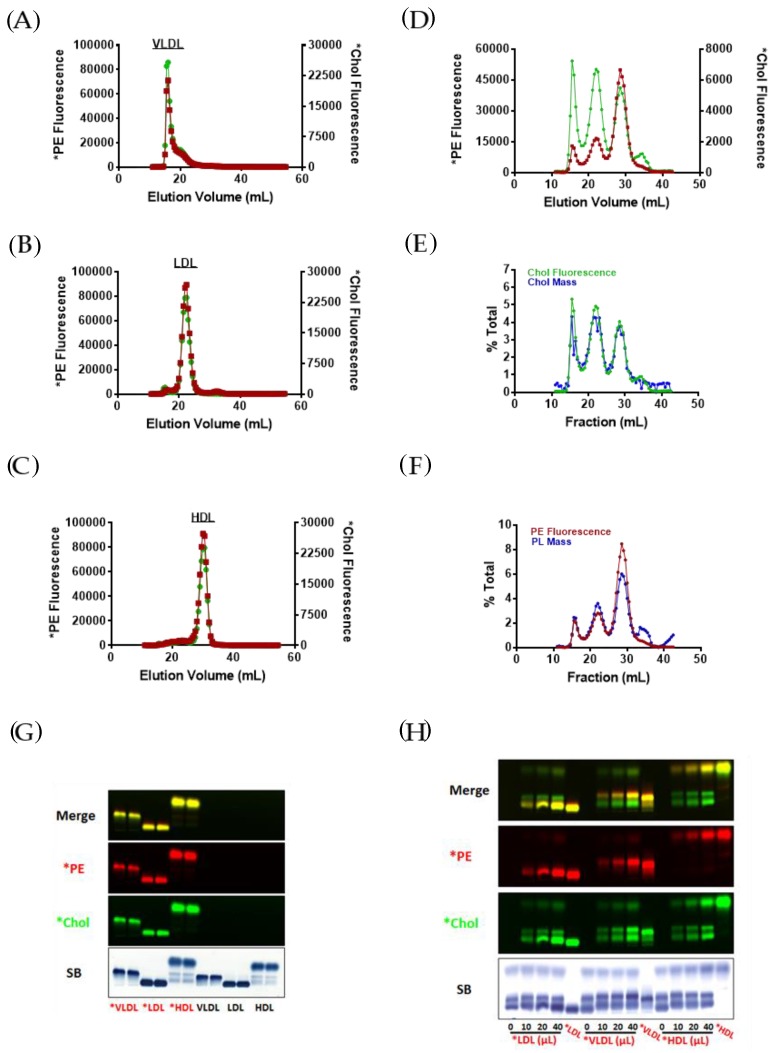
Incorporation of fluorescent phosphatidylethanolamine (PE) and cholesterol into human plasma lipoproteins. FPLC analysis of fluorescent PE (*PE) and cholesterol (*Chol) dual-labeled isolated human lipoproteins. (**A**) VLDL (**B**) LDL (**C**) HDL. Red line: *PE fluorescence; green line: *Chol fluorescence. FPLC analysis of human plasma labeled with both *PE and *Chol. (**D**) Distribution of *PE and *Chol in pooled human serum lipoproteins separated by FPLC. Red line: *PE fluorescence; green line: *Chol fluorescence. (**E**) Comparison of *Chol fluorescence (green line; % of total *Chol fluorescence) with cholesterol mass (blue line; % of total cholesterol mass). (**F**) Comparison of *PE fluorescence (red line; % of total *PE fluorescence) with phospholipid mass (blue line; % of total phospholipid mass). (**G**) Human plasma-derived *VLDL, *LDL, and *HDL labeled with both *PE (red) and *Chol (green) as well as unlabeled VLDL, LDL, and HDL were separated by agarose gel electrophoresis (45 min). (**H**) Agarose gel electrophoresis of human plasma (HP) incubated with *PE (green) and *Chol (red) dual-labeled isolated human *VLDL, *LDL, or *HDL, as described in “Materials and Methods.” After fluorescence was imaged (**G**,**H**), gels were stained with Sudan Black (SB) and re-scanned (*n* = 3).

We first assessed the effect of labeling isolated lipoprotein subfractions with trace amounts of both fluorescent PE (*PE) and cholesterol (*Chol) by FPLC lipoprotein analyses and found that the typical elution profiles for VLDL, LDL, and HDL were unaltered by the labeling process (Figure 1A–C). The distribution of *Chol and *PE among lipoproteins detected by agarose gel electrophoresis (Figure 1G) was similar to that observed by FPLC analysis (Figure 1A–C). The electrophoretic mobility of VLDL and HDL fractions on agarose gels was increased by dual *PE and *Chol labeling, most likely due to the presence of the positively charged fluorophore PE on the lipoprotein particles (Figure 1G). The migration of LDL was not evidently affected; however, LDL migration on agarose gels is very slow so that any change imposed by the fluorophores on *PE and *Chol may be difficult to detect.

We next tested whether labeling of whole human plasma with *PE and *Chol would alter the FPLC elution profile of plasma lipoproteins, and, if incorporation of the fluorescent lipids into lipoproteins would be proportional to lipid mass. As before, the plasma lipoprotein phospholipid and cholesterol FPLC elution profiles were unaffected by labeling with either *PE or *Chol, but the pattern of incorporation of *PE and *Chol incorporation into plasma lipoproteins differed (Figure 1D). The incorporation of *PE and *Chol into lipoproteins, however, was proportional to lipoprotein cholesterol and phospholipid, respectively (Figure 1E,F). Thus, the differing patterns of incorporation of *PE and *Chol into lipoproteins reflects the different phospholipid and cholesterol composition of VLDL, LDL and HDL particles.

Finally, we incubated increasing volumes of dual *PE- and *Chol-labeled isolated VLDL, LDL and HDL with whole pooled human plasma to assess the transferability of *PE and *Chol between lipoproteins. As shown in Figure 1H, agarose gel analysis reveals that *PE remained associated with the original lipoprotein particle regardless of the amount of lipoprotein added. This finding suggests that *PE does not exchange between plasma lipoproteins and can serve as a lipoprotein reference marker. In marked contrast, *Chol derived from the added lipoprotein readily transferred to the other plasma lipoproteins in a dose-dependent manner and in accordance with Sudan Black staining (Figure 1H).

**Figure 2:**
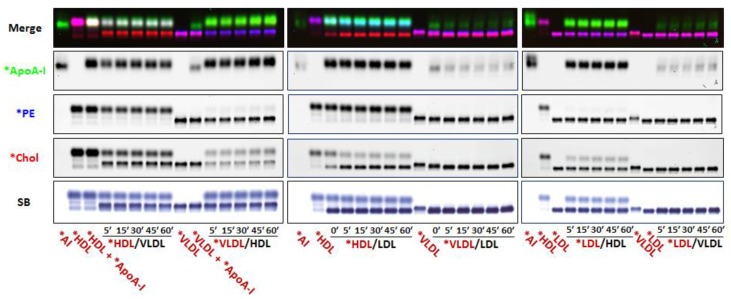
Fluorescent Alexa647-tagged apoA-I binds specifically to HDL. Fluorescence agarose gel electrophoresis. Left panel: Alexa 647-tagged-ApoA-I (*ApoA-I; green) was incubated with equal volumes of *HDL labeled with both *PE (blue) and *Chol (red) and unlabeled VLDL, and conversely, with equal volumes of *VLDL labeled with both *PE and *Chol and unlabeled HDL for the indicated times (mins). Middle panel: *ApoA-I was incubated with either equal volumes of *HDL or *VLDL and unlabeled LDL for the indicated times (mins). Right panel: *ApoA-I was incubated with equal volumes of *LDL and either unlabeled HDL or VLDL, for the indicated times (mins). Gel running times were 45 min. Note that (i) the free *apoA-I (*AI) band runs just below *HDL, (ii) *apoA-I binds to HDL but not to VLDL or LDL, (iii) *PE remains associated with the originally fluorescent labeled lipoprotein and, (iv) *Chol rapidly exchanges between labeled and unlabeled lipoproteins (*n* = 3).

In the next series of experiments, we co-incubated dual *PE- and *Chol-labeled isolated lipoproteins and Alexa647-ApoA-I (*apoA-I) with unlabeled lipoproteins to determine if (i) *PE would remain with the original lipoprotein; (ii) *Chol would exchange between labeled and unlabeled lipoproteins, and (iii) if Alexa647-tagged apoA-I would specifically bind to HDL. We have previously shown that apoA-I functionality, as defined by apoA-I-mediated cellular cholesterol efflux, is not altered when apoA-I is tagged with an Alexa fluorophore [28]. *ApoA-I, *PE and *Chol fluorescence was monitored by agarose gel electrophoresis. *ApoA-I bound specifically to HDL, confirming that the fluorescent tags on apoA-I or lipids did not alter apoA-I binding to HDL nor induce non-specific binding to other lipoproteins either in the presence or absence of HDL (Figure 2). In these experiments, we ran agarose gels for 45 min to avoid smearing of the *apoA-I bands on the gel. Consistent with our observation above (Figure 1H), where fluorescent lipoproteins were incubated with unlabeled pooled human plasma, *PE remained associated with the original fluorescent-labeled lipoprotein (Figure 2), whereas *Chol rapidly exchanged (within 5 min) between fluorescent lipid-labeled and unlabeled lipoproteins. 

**Figure 3:**
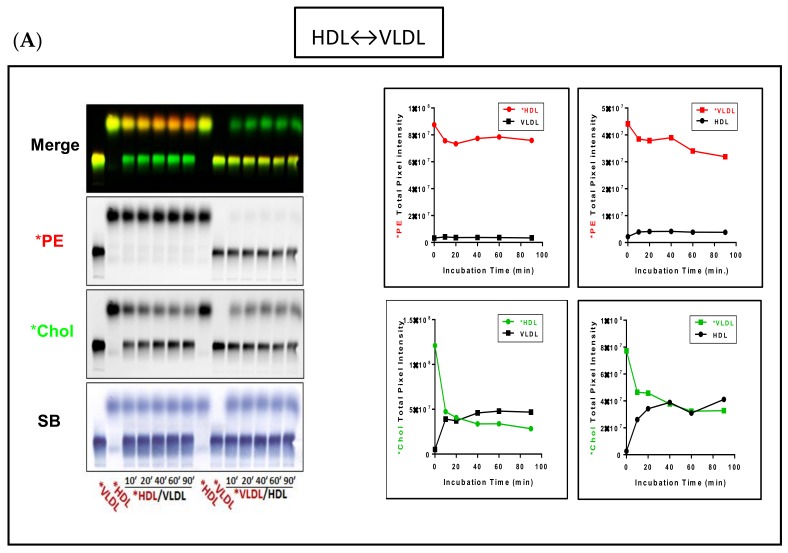

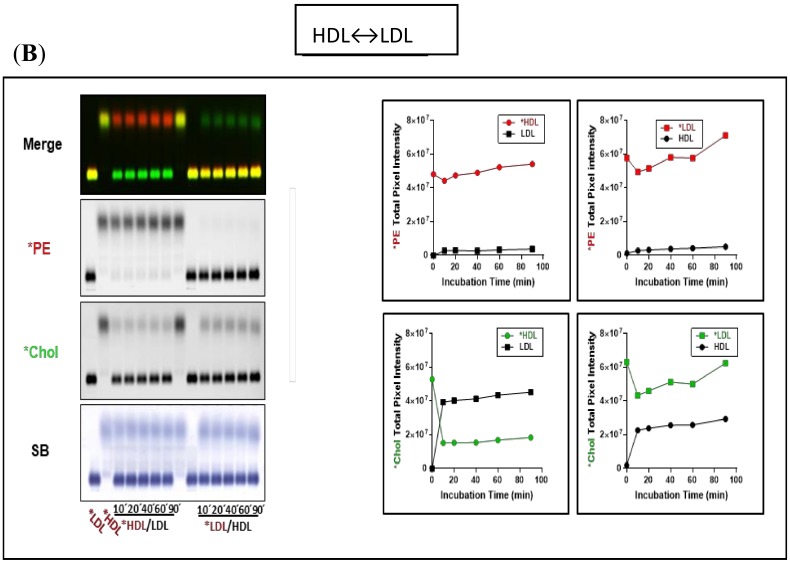

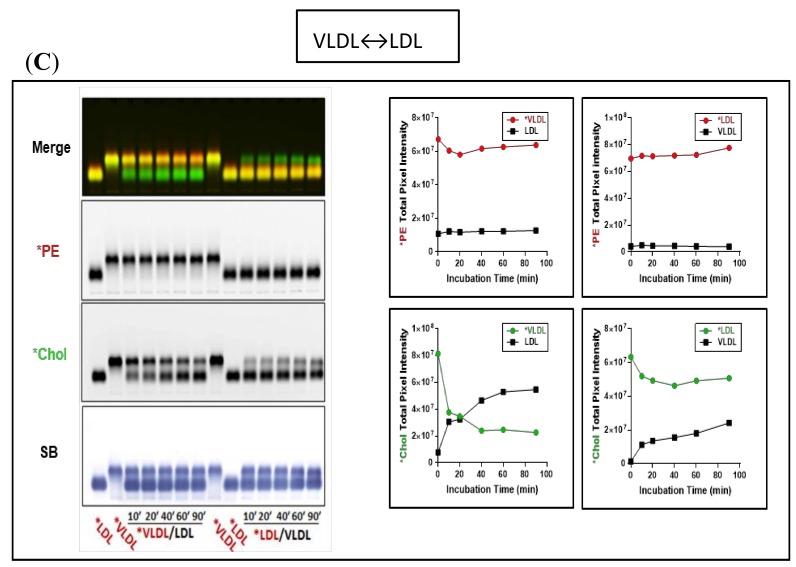
Fluorescent cholesterol exchange between lipoproteins. Fluorescence agarose gel electrophoresis. Gel running times, 90 min. (**A**) Cholesterol exchange between HDL and VLDL. Equal volumes of dual fluorescent *PE- and *Chol-labeled HDL (*HDL) and unlabeled VLDL (left side of gel), and conversely, *VLDL with unlabeled HDL (right side of gel), were incubated for the indicated times (mins). Unmixed *HDL and *VLDL lanes are indicated by red font. (**B**) Cholesterol exchange between HDL and LDL. Equal volumes of *HDL and unlabeled LDL (right side of gel), and conversely, *LDL with unlabeled HDL (left side of gel), were incubated for the indicated times (mins). Unmixed *LDL and *HDL lanes are indicated by red font. (**C**) Cholesterol exchange between VLDL and LDL. Equal volumes of *VLDL and unlabeled LDL (right side of gel), and conversely, *LDL with unlabeled VLDL (left side of gel), were incubated for the indicated times (mins). Unmixed *LDL and *VLDL lanes are indicated by red font. Quantification of total pixel intensity of original labeled lipoprotein for *PE (red line) and *Chol (green line) fluorescence and of unlabeled lipoproteins (black lines) for (**A**–**C**) is shown on the right side of each panel. Note that *PE remained associated with the originally labeled lipoprotein in all cases (*n* = 3).

To better assess *Chol exchange between lipoproteins, we conducted labeled and unlabeled lipoprotein mixing experiments and ran the agarose gels for 90 min to enhance resolution of the VLDL and LDL bands (Figure 3). These studies again demonstrate that *PE remains associated with the original fluorescent labeled lipoprotein, and further, that *Chol exchange between lipoproteins is both time- and dose-dependent.

As seen in Figure 3A, VLDL and HDL cholesterol rapidly equilibrated (within ~10–20 min). In marked contrast, *HDL rapidly transferred (10 min) about 60% of its *Chol to LDL, whereas *LDL transferred only about 30% of its *Chol to HDL (Figure 3B). Since there was a net transfer of *Chol to LDL from HDL, and retention of *Chol by LDL, LDL seems to have a higher apparent affinity for cholesterol than HDL does. Similarly, as seen in Figure 3C, *VLDL rapidly donated (10 min) 60% of its *Chol to LDL and conversely, *LDL retained about 85% of its *Chol, suggesting that LDL also has a higher apparent affinity for cholesterol than VLDL. Dose response studies provided further evidence that LDL has a higher apparent affinity for *Chol than VLDL (Supplemental Figure S1A). However, when *Chol transfer between LDL and VLDL was evaluated based on the acceptor/donor ratio of unesterified cholesterol mass (Supplemental Figure S1B,C), there appeared to be a net transfer of LDL to VLDL simply because of the increased pool size of unesterified cholesterol in the LDL volumes used.

### 3.2. Fluorescent PE Can Be Used to Monitor Lipoprotein Remodeling

We have previously demonstrated that incubation of *PE-labeled synthetic lipoprotein-X multilamellar vesicles (LpX) with apoA-I generates a pre-β HDL-like particle that in the presence of LCAT is converted into an HDL-like particle both in vitro and in vivo [14]. Unlike typical lipoproteins, LpX migrates toward the cathode during agarose gel electrophoresis [29], and thus can be readily detected.

**Figure 4:**
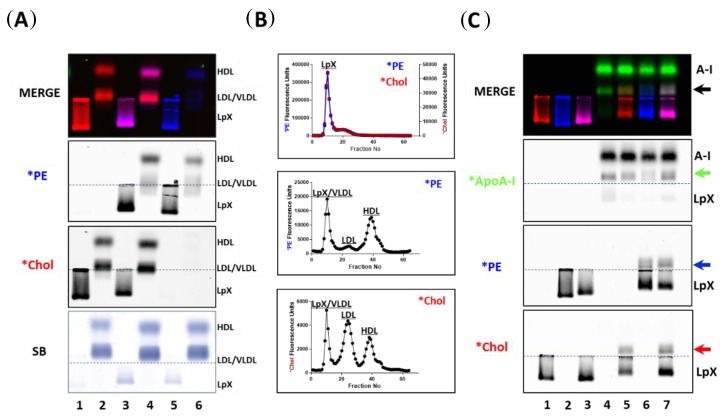
ApoA-I-mediated lipoprotein-X (LpX) remodeling and transfer of LpX cholesterol to human plasma lipoproteins. (**A**) Pooled human plasma (HP) was incubated with fluorescent lipid-tagged synthetic LpX overnight. Lane 1: LpX labeled with *Chol; Lane 2: Human plasma (HP) incubated with LpX labeled with *Chol; Lane 3: LpX labeled with both *PE and *Chol; Lane 4: HP incubated with LpX labeled with both *PE and *Chol; Lane 5: LpX labeled with *PE; Lane 6: HP incubated with LpX labeled with fluorescent PE. (**B**) FPLC analyses of synthetic fluorescent lipid-tagged LpX before (upper panel) and after (middle and lower panels) incubation with pooled human plasma. In these studies, plasma was incubated with excess LpX. (**C**) Unlabeled or fluorescent lipid-labeled LpX was incubated with Alexa647-tagged ApoA-I overnight. Lane 1: LpX labeled with *Chol; Lane 2: LpX labeled with *PE; Lane 3: LpX labeled with both *PE and *Chol; Lane 4: Unlabeled LpX + Alexa647-tagged ApoA-I; Lane 5: LpX labeled with *Chol + Alexa647-tagged ApoA-I; Lane 6: LpX labeled with *PE + Alexa647-tagged ApoA-I; Lane 7: LpX labeled with both *PE and *Chol+ Alexa647-tagged ApoA-I. Colored arrows indicate newly-formed pre-β HDL-like particles. The reaction mixture components were separated by agarose gel electrophoresis for 45 min and then, fluorescence was imaged as described. The origin on agarose gels is indicated by dashed blue lines in (**A**,**C**) (*n* = 3).

For the present studies, we synthesized multilamellar synthetic LpX particles [14] that contained trace amounts of *PE, *Chol, or both, and then incubated the fluorescent LpX with human plasma (Figure 4A,B). Nearly all the fluorescent LpX was consumed following incubation with human plasma (Figure 4A). LpX-derived *PE associated specifically with HDL (Figure 4A,B). The partitioning of LpX-derived *Chol onto lipoproteins (Figure 4A,B) was similar to that seen above (Figure 1H), indicating that LpX-associated *Chol equilibrates among lipoproteins based on their cholesterol content.

We then incubated single or dual fluorescent lipid-labeled LpX with excess fluorescent-tagged or non-tagged apoA-I, to test if apoA-I would remove lipids from LpX, and, if the fluorescent tags would interfere with this process. As shown in Figure 3C, *apoA-I did indeed remodel LpX to form a new lipoprotein particle containing *PE, *Chol and *apoA-I that migrated to the pre-β HDL position on the gel, similar to our previous study [14]. The remodeling of LpX by *apoA-I did not appear to be perturbed by the presence of the fluorophores on either of the lipids.

### 3.3. Fluorescent Lipid Transfer to Lipoproteins from Substrate-Bound Lipid Donor Membranes

In the preceding studies, we directly labeled lipoproteins with fluorescent lipids and apoA-I in order to monitor cholesterol exchange between lipoproteins and remodeling of lipoproteins. Given the interest in cellular cholesterol efflux as a marker of HDL functionality [30], fluorescent lipid-labeled donor membranes that mimic cell membrane lipid surface structure or lipid composition, and are readily separable from acceptor membranes, could serve as an additional useful model system to study lipid transfer from a donor particle to plasma HDL.

To this end, we developed a simple method to assess fluorescent PE and cholesterol transfer from substrate-bound donor membranes to lipoproteins. We used DMPC:cholesterol lipid donor membranes with a composition similar to synthetic LpX, since we have shown above that (i) *apoA-I removes *PE and *Chol from synthetic LpX to form a pre-β HDL-like particle; (ii) LpX transfers both *PE and *Chol to HDL in an apoA-I-dependent manner, and (ii) *Chol to VLDL and LDL by exchange. We coated calcium silicate hydrate crystals (Lipid Removal Adsorbent (LRA)), which readily adsorb lipids, with DMPC, cholesterol and trace amounts of *PE and *Chol. The donor lipid membrane preparations were readily separated from the lipoprotein acceptor particles by a simple low speed centrifugation step, which pellets the donor, but not the acceptor particles.

**Figure 5:**
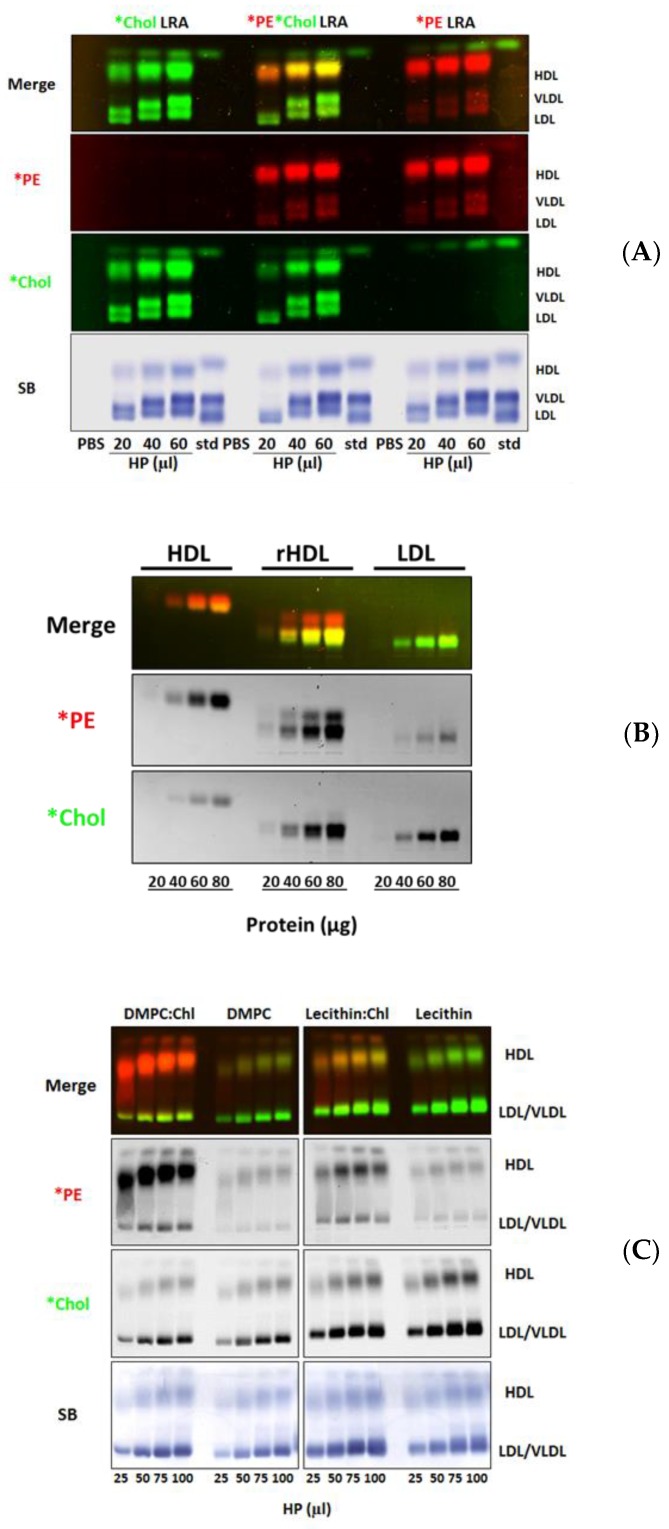
Transfer of Lipid Removal Adsorbent (LRA) lipid particle fluorescent PE and cholesterol to whole human plasma, isolated human HDL and LDL and, reconstituted HDL. (**A**) Agarose gel electrophoresis of 0, 20, 40, or 60 µL of human plasma incubated with 50 µL of LRA lipid particles labeled with either *Chol, *PE, or both, for 4 h. (**B**) Agarose gel electrophoresis of HDL, reconstituted HDL (rHDL), or LDL (0, 20, 40, 60, or 80 µg protein) incubated with 60 µL of *PE- and *Chol-labeled LRA lipid particles for 4 h. (**C**) Agarose gel electrophoresis of human plasma incubated with *PE- and *Chol-labeled LRA lipid particles containing either DMPC:Chol (4:1), DPMC alone, egg lecithin:Chol (4:1), or, egg lecithin alone for 1 h (*n* = 3).

To assess the potential specificity of the acquisition of lipid-coated LRA *PE by HDL, as well as possible interactions between the Lissamine Rhodamine B and Bodipy fluorophores, we incubated singly or doubly-labeled fluorescent *PE and *Chol lipid-coated LRA with normal human plasma. LRA-derived *PE robustly transferred to plasma HDL with little or no transfer to VLDL or LDL (Figure 5A), consistent with the previously observed HDL-specific uptake of LpX-derived *PE in whole human plasma (Figure 4A). As seen previously using synthetic LpX (Figure 4A), lipid-coated LRA transferred *Chol to all lipoproteins (Figure 5A). Transfer of both lipid-coated LRA *PE or *Chol to lipoproteins occurred in a dose-dependent manner. The distribution of *PE and *Chol that transferred from singly- or doubly-labeled *PE and/or *Chol lipid-coated LRA to plasma lipids was nearly identical, indicating that Lissamine Rhodamine B and Bodipy fluorescence were not altered when the two fluorophores were present together on lipoprotein particles.

rHDL, an avid cholesterol acceptor, is a synthetic pre-ß-like HDL particle that is made by reconstituting lipid-free apoA-I with phosphatidylcholine [31]. Donor particle-derived *PE robustly transferred to isolated HDL and rHDL but not to LDL (Figure 5B), consistent with the previously observed HDL-specific uptake of LpX-derived *PE (Figure 4A,B) and lipid-coated LRA donor particle-derived *PE (Figure 5A) in whole human plasma. Transfer of both donor lipid particle *PE and *Chol to lipoproteins occurred in a dose-dependent manner (Figure 5). Interestingly, rHDL served as an avid cholesterol acceptor (Figure 5B). Fluorescent agarose gel electrophoresis revealed that the rHDL formed two bands: (1) a slower migrating band that acquired both *Chol and *PE from lipid donor LRA particles, and (2) a faster migrating band that acquired only *PE. Thus, the latter band was only revealed by its uptake of *PE. Based on total *PE and *Chol fluorescence intensity (Figure 5B), and their respective fluorescence/mass ratios (Supplemental Figure S2), we calculated that 3 moles of *Chol transferred per mole of *PE from lipid LRA particles to recombinant HDL (rHDL).

We examined the effect of altering the phospholipid composition and cholesterol content of LRA donor particles on the transfer of fluorescent PE and cholesterol to human plasma lipoproteins. As seen in Figure 5C, in the absence of cholesterol, apoA-I-mediated transfer of *PE was markedly reduced, independent of the phospholipid composition (DMPC vs. egg lecithin). However, with the addition of cholesterol to phospholipid donor particles, specific *PE transfer to plasma HDL was markedly increased, and was dependent on the phospholipid composition (DMPC > egg lecithin). This finding suggests that cholesterol may alter the organization of phospholipids on the LRA particle surface, rendering *PE more available for apoA-I-mediated efflux. Transfer of *Chol from donor LRA particles to all plasma lipoproteins by passive exchange, on the other hand, was solely dependent on donor LRA phospholipid composition. Based on these findings, DMPC-containing LRA donor lipid particles were routinely used.

**Figure 6:**
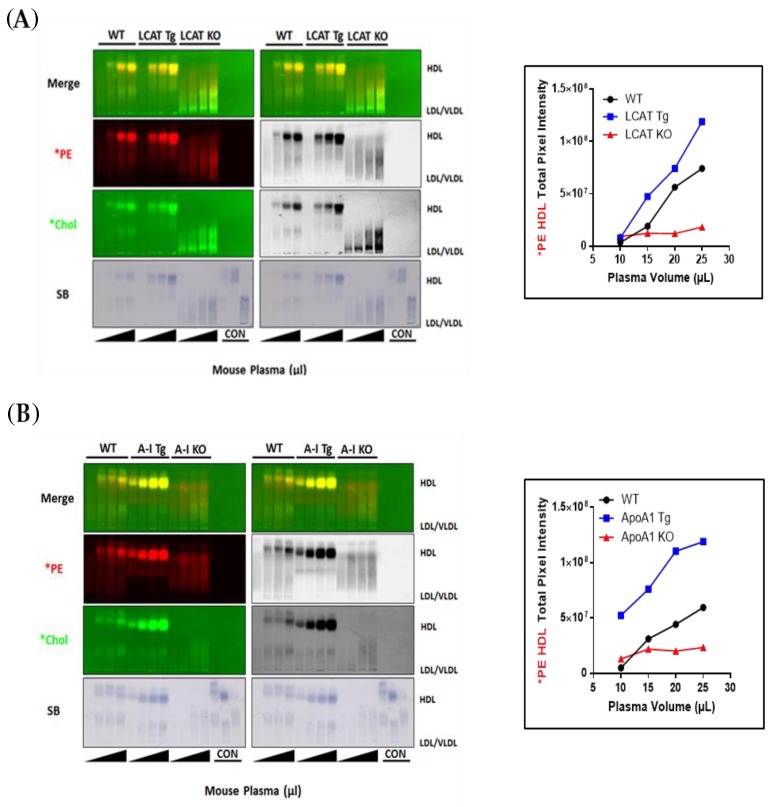
Transfer of LRA lipid particle fluorescent PE and cholesterol to wild-type and LCAT- and apoA-I-Tg and -KO mouse plasma. (**A**) Left: Agarose gel electrophoresis of 10, 15, 20, or 25 µL wild-type, LCAT-Tg and LCAT-KO mouse plasma incubated with 50 µL *PE and *Chol-labeled LRA lipid particles for 1 h at 37 °C with mixing. Right: Quantitative analysis of gel band *PE HDL fluorescence. Note that dose-dependent *PE and *Chol transfer to HDL is increased in LCAT-Tg mouse plasma and absent in LCAT-KO mouse which lack plasma HDL and dose-dependent *Chol transfer to LDL is increased in LCAT-KO plasma, which contains increased LDL. (**B**) Agarose gel electrophoresis of 10, 15, 20, or 25 µL wild-type, apoA-I-Tg and apoA-I-KO mouse plasma incubated with 50 µL *PE- and *Chol-labeled LRA lipid particles for 1 h at 37 °C with mixing. Right: Quantitative analysis of gel band *PE HDL fluorescence. Note that dose-dependent *PE and *Chol transfer to HDL is markedly increased in apoA-I-Tg mouse plasma and absent in ApoA-I -KO mouse which lack plasma HDL (*n* = 3).

To directly confirm the role of HDL and apoA-I in mediating *PE transfer from donor LRA particles to plasma HDL, we compared *PE transfer from fluorescent lipid-coated LRA particles to plasma lipoproteins from wild-type and genetically engineered knock-out and transgenic LCAT and apoA-I mice. We have previously shown that plasma HDL concentrations are increased in LCAT-Tg mouse plasma [23] and are increased to an even greater extent in apoA-I Tg mice [32]. As can be seen in Figure 6A, as compared to wild-type mouse plasma, *PE transfer to HDL was increased to LCAT-Tg mouse plasma HDL, in a plasma dose-dependent manner, and was absent in LCAT-KO mice, which lack HDL. These findings demonstrate that lipid donor particle *PE plasma uptake is HDL- dependent, and proportional to the plasma HDL concentration. ApoA-I Tg mice have markedly increased levels of plasma HDL. As can be seen in Figure 6B, compared to wild-type mouse plasma, accordingly, LRA donor particle *PE transfer to apoA-I Tg mouse plasma HDL was markedly increased and was also plasma dose-dependent, and absent in apoA-I KO mice, which lack HDL. These findings demonstrate that LRA donor particle *PE transfer to mouse plasma is apoA-I-dependent.

**Figure 7:**
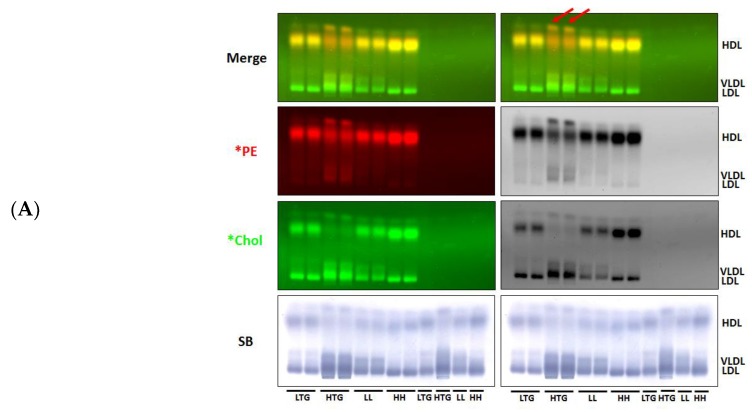

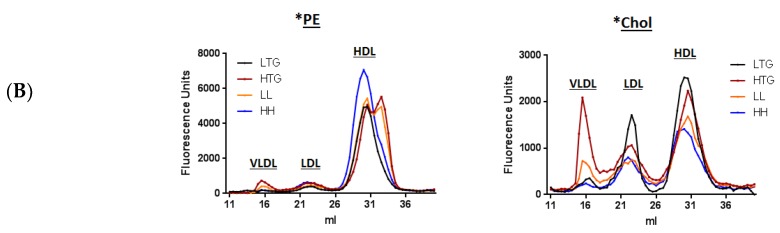
Transfer of LRA lipid particle fluorescent PE and cholesterol to pooled human plasma samples with different LDL, HDL and triglyceride contents. (**A**) Agarose gel electrophoresis of different pooled human plasma samples incubated with *PE- and *Chol-labeled LRA lipid particles. Lanes 1,2: *LTG; Lanes 3,4: *HTG; Lanes 5,6: *LL; Lanes 7,8: *HH; Lanes 9–12: unlabeled LTG, HTG, LL, HH. Red arrows indicate uppermost band, which is enriched with both albumin and apoA-I. (**B**) Distribution of LRA lipid particle-derived *PE and *Chol in FPLC fractions of pooled human plasma samples. LTG: LowTriglyceride TG; HTG: High TG; LL: Low LDL; HH: High HDL (*n* = 3).

We next assessed the use of lipid-coated LRA in evaluating the transfer of fluorescent PE and cholesterol to pooled human plasma lipoprotein samples containing varying amounts of LDL, HDL, and triglycerides (Supplemental Table S1). As assessed by agarose gel analysis and FPLC, *PE transferred from donor particles to HDL, but little, if any, *PE transferred to VLDL or LDL (Figure 7A,B). This finding is consistent with our previous observations that donor LRA particle *PE is actively incorporated into human plasma HDL (Figure 5A). HDL *PE efflux in the different pooled samples appeared to correlate with their plasma apoA-I content (Supplemental Figure S3), consistent with the apoA-I dependence observed in our mouse plasma studies (Figure 6). In contrast to *PE, *Chol transferred from lipid-coated LRA to all lipoproteins (Figure 7A,B), in a similar manner to our studies above. FPLC analysis revealed that the different pooled human plasma samples displayed unique patterns of uptake of both *PE and *Chol (Figure 7B).

This methodology also allowed us to identify a band above the HDL band on agarose gels that acquired both *PE and *Chol (Figure 7A,B). We conducted in-gel proteomic analysis of these two bands, which to our knowledge, is a method that has not been previously reported. Proteomic analysis of the proteins that were identified in the HDL band vs. the established HDL proteome [33] revealed that all but one of the proteins (coagulation factor 10 (FA10)) were bona fide HDL binding proteins, thus validating the in-gel mass spectrometric proteomic analysis. Mass spectrometric proteomic analysis revealed the uppermost band in the HTG sample was markedly enriched with apoA-I and apoA-II, but not other HDL-associated proteins. Since we have shown robust uptake of both *PE and *Chol by rHDL from donor particles (Figure 4), this finding suggests that other HDL species (potentially pre-α-HDL [34]) present in the uppermost band likely acquire fluorescent PE and cholesterol from the donor particles.

Mass spectrometric proteomic analysis revealed that the uppermost band in the HTG sample was also enriched with albumin (Table 1). Albumin and total protein plasma concentrations were within the normal range for all four samples (4.33 and 7.36, 3.55 and 6.61, 4.23 and 7.41, and, 4.24 and 7.15, mg/dL and g/dL, respectively, for LTG, HTG, LL, HH) and did not correlate with the intensity of either *PE or *Chol labeling of the uppermost band. Based on these findings, we then assessed if albumin itself can serve as an acceptor for lipid-coated LRA *PE or *Chol and found that both *PE and *Chol transfers from donor lipid LRA particles to human serum albumin in vitro (Supplemental Figure S4). As has been previously suggested [35], albumin may play a role in shuttling both phospholipids and cholesterol between lipoproteins and other lipid acceptors and may also serve as a reservoir for lipoprotein-derived lipids. Taken together, these findings suggest that apoA-I (potentially pre-α-HDL) and albumin observed in the uppermost band in human plasma may acquire both fluorescent phospholipids and cholesterol either directly from donor LRA particles, and/or indirectly, from other plasma lipoproteins.

**Figure 8:**
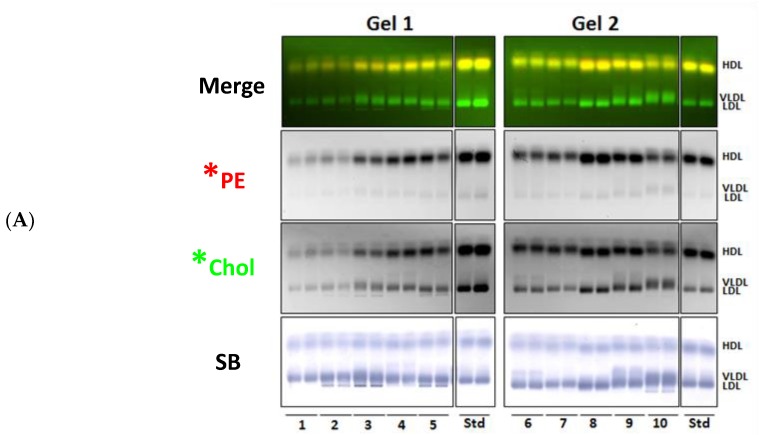

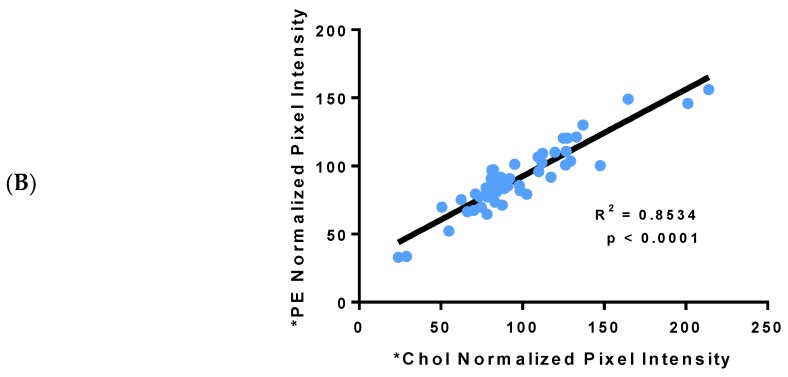
Transfer of LRA lipid particle fluorescent PE and cholesterol to HDL in different individual human plasma samples. Fifty human plasma samples were incubated with lipid-coated LRA labeled with *PE and *Chol. (**A**) Fluorescent plasma lipoproteins were separated by agarose gel electrophoresis and *PE and *Chol fluorescence imaged as described. Imaging conditions for each fluorophore were standardized to 70% of maximal intensity using the HDL band in the reference standard. Two representative gels (out of 10) are shown. (**B**) The total pixel intensity of HDL *PE and *Chol bands were quantified as described for all fifty samples and normalized with respect to the reference standard on the gels (unknown HDL *PE/ standard HDL *PE; unknown HDL *Chol/standard HDL *Chol). Note that HDL *PE and *Chol fluorescence are highly correlated (*n* = 2).

Lastly, we assessed the transfer of *PE and *Chol from LRA lipid donor particles to a set of human plasma samples from fifty individuals (Figure 8). Agarose gel electrophoresis reproduced the results observed in Figure 8A, namely that *PE transferred only to HDL (and variably to the albumin band) and that *Chol transferred to VLDL, LDL, and HDL. The intensity of HDL *PE and *Chol fluorescence in these fifty samples was highly correlated (Figure 8B; R^2^ = 0.8534; *p* < 0.0001), suggesting that the mole ratio of phospholipid and cholesterol apoA-I-mediated transfer from LRA to HDL donor lipid particles is highly conserved.

## 4. Discussion

Our studies have established that head-group-tagged fluorescent PE can serve as a reference marker for lipoprotein particles. Fluorescent PE incorporation into plasma lipoproteins was proportional to phospholipid mass and *PE remained associated with lipoproteins even after incubation with either whole unlabeled plasma (Figure 1), or, with unlabeled VLDL, LDL, or HDL. In marked contrast, fluorescent cholesterol, which similarly incorporated into plasma lipoproteins proportional to cholesterol mass, readily exchanged between lipoproteins in whole or sub-fractionated plasma lipoproteins. Exchange of *Chol between isolated subfractions of VLDL, LDL and, HDL was rapid and both time- and dose-dependent. While it has long been known that cholesterol rapidly equilibrates between lipoproteins [36], little is known about the relative affinity of different lipoproteins for cholesterol. LDL typically has the highest content of unesterified cholesterol (per mg protein), and in vivo, LDL has been shown to serve as a plasma reservoir, with a large capacity to hold cholesterol and thus act as a cholesterol sink [37]. Consistent with these previous observations, we found that LDL served as a *Chol sink when the LDL unesterified cholesterol content exceeded that of VLDL or HDL.

We further demonstrated that fluorescent-tagged apoA-I, an exchangeable apolipoprotein, bound specifically to HDL. A considerable number of well-characterized apolipoproteins are known to associate with lipoproteins, and recent proteomic analyses of VLDL, LDL, and HDL have identified a large number of additional proteins that reside on the surface of lipoprotein particles, most notably HDL. In addition, a number of peptides, including mimetic peptides, that associate with HDL are being developed as therapeutic agents to improve lipoprotein functionality. The lipoprotein binding properties and exchangeability of all the exchangeable apolipoproteins and peptides that can be fluorescently labeled and maintain their functionality are potentially amenable to study using the methods we have described.

Having established that fluorescent PE can serve as a lipoprotein marker and that fluorescent cholesterol readily exchanges between lipoproteins, we then demonstrated the utility of using both fluorescent lipids, as well as fluorescent apoA-I, to monitor lipoprotein remodeling events. Based on the finding that apoA-I removed both fluorescent PE and cholesterol from LpX particles, we developed a novel model system by coating LRA donor particles with LpX-like lipids in order to study lipid transfer to lipoproteins. Our studies using LCAT- and apoA-I KO and Tg mouse plasma confirmed that lipid donor particle *PE transfer is HDL-specific and apoA-I-dependent.

**Figure 9:**
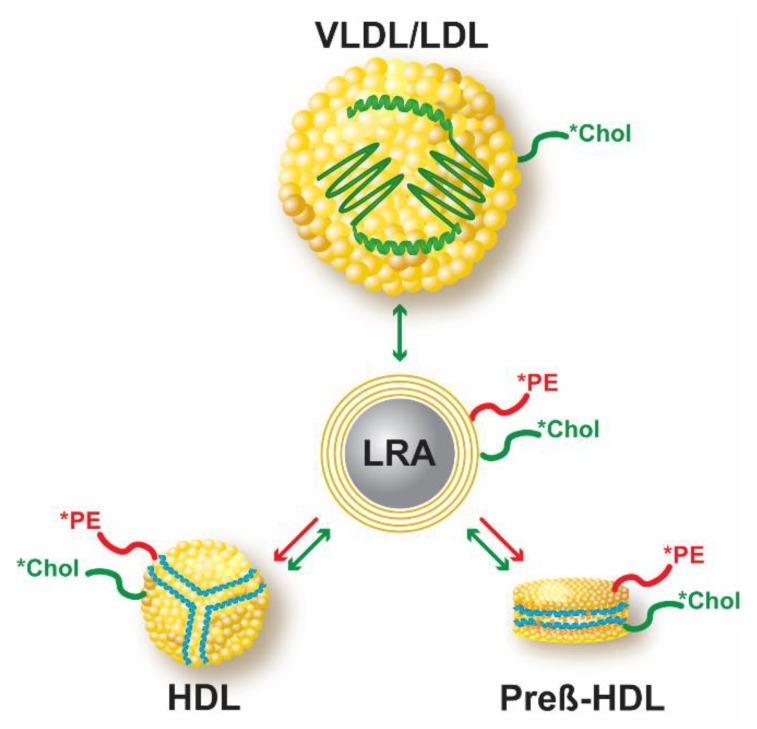
Model of apoA-I-mediated transfer of lipid-coated LRA *PE to HDL. Lipid-coated LRA particles containing fluorescent cholesterol (*Chol; green) and PE (*PE; red) can be used to monitor both the active removal of fluorescent PE, by HDL-apoA-I-mediated membrane solubilization as well as passive exchange of fluorescent cholesterol to (and between) plasma lipoproteins. ApoA-I on the surface of HDL and pre-β-HDL is represented by blue wavy lines and apo-B on the surface of VLDL and LDL as a green wavy line.

The pattern of *PE and *Chol transfer from LRA lipid particles to lipoproteins in pooled human plasma samples differed with plasma lipoprotein levels. This finding suggests that apoA-I-mediated phospholipid uptake by HDL (as well as cholesterol exchange between lipoproteins) differs according to the intrinsic properties of the particular lipoprotein species that are present in the plasma sample. Indeed, HDL-mediated lipid efflux was recently shown to be dependent on apoA-I exchangeability [8,9]. ApoA-I can dissociate from HDL particles and then associate with another HDL particle, or alternatively, insert into the plasma membrane of a cell. The acquisition of phospholipids by apoA-I, unlike cholesterol, does not involve phospholipid desorption, but rather occurs by active solubilization of an ABCA1-generated plasma membrane lipid domain by inserted apoA-I; lipidation of membrane-inserted apoA-I alters the protein’s conformation, resulting in the dissociation of lipidated apoA-I from the plasma membrane. Presumably, the transfer of *PE from either donor LpX MLVs or lipid-coated LRA particles to apoA-I occurs by a similar process involving insertion of HDL-derived apoA-I into the lipid surface followed by dissociation of lipidated apoA-I from the donor lipid particle (Figure 9). ApoA-I may also acquire some *Chol during this process, but also likely acquires fluorescent cholesterol by equilibration of donor and acceptor cholesterol pools via passive exchange.

Albumin has been shown to shuttle cholesterol between lipoproteins and from lipoproteins to red blood cells [35]. Our studies provide further evidence that albumin may serve as a conduit and perhaps as a reservoir for mobilized plasma lipoprotein cholesterol and that albumin also mobilizes plasma lipoprotein phospholipid. We observed that the albumin present in human plasma samples with different lipoprotein profiles harbored different amounts of cholesterol and phospholipid. Thus, our methodology revealed that human plasma lipoproteins may have variable amounts of surface phospholipids and cholesterol that can be mobilized by albumin. Additional studies are needed to confirm these intriguing findings. Moreover, our proteomic analysis revealed an albumin-enriched band above HDL that contained both *PE and *Chol that is also highly enriched with both apoA-I and apoA-II. Given that we also showed that rHDL avidly removes both *PE and *Chol from LRA donor lipid particles, it is likely that plasma α-lipoproteins also engage in this lipid efflux.

Recently, assays to monitor cholesterol uptake by plasma HDL from donor lipid particles labeled with Bodipy-cholesterol as alternative assays for cell-based ^3^H-cholesterol efflux assays have been developed [17,18]. These assays monitor the equilibration of fluorescent cholesterol between donor particles and HDL surface pools of cholesterol by passive exchange, and, have been shown to correlate with cellular cholesterol efflux capacity, as well as cardiovascular events. In our assay, HDL-mediated removal of a fluorescent-labeled phospholipid from a donor particle occurs by an active mechanism that is dependent on apoA-I functionality. Importantly, our evaluation of fifty human plasma samples revealed that *PE and *Chol efflux from LRA donor lipid particles was highly correlated, thereby confirming that *PE efflux can serve as a marker for apoA-I-HDL-mediated efflux. Our studies have established that cell-free *PE efflux can monitor apoA-I functionality and, therefore, may provide a platform to identify dysfunctional HDL. A high-throughput version of this assay may be used in future studies to determine whether cell-free *PE efflux to HDL correlates with cell-mediated cholesterol efflux and/or other biomarkers related to cardiovascular disease or with clinical outcomes.

## 5. Conclusions

Using a head-group-tagged fluorescent PE as a lipoprotein marker, we have demonstrated the utility and diverse applicability of simple, sensitive and robust methods to monitor lipoprotein metabolism and remodeling, as well as lipid and apolipoprotein exchange in vitro using fluorescent cholesterol and apoA-I. This methodology advantageously allows for multi-modal lipid and lipoprotein fluorescence-based analyses including, but not limited to, electrophoresis, FPLC, fluorimetry, and confocal microscopy.

## 6. Patents

E.B.N, M.S. (Masaki Sato) and, A.R.T. have submitted the patent application E-085-2019-1-US-01 based in part on the work reported in this manuscript.

## Figures and Tables

**Table 1:** Proteomic Analysis of HTG Pooled Human Plasma ALB and HDL Bands.

Identified Proteins (30)	Accession Number	Molecular Weight	ALB (*TSC)	HDL (*TSC)
Serum albumin	ALBU	69 kDa	938	421
Apolipoprotein A-I	APOA1	31 kDa	58	4
Vitamin D-binding protein	VTDB	53 kDa	0	50
Prothrombin	THRB	70 kDa	0	49
Inter-alpha-trypsin inhibitor heavy chain H2	ITIH2	106 kDa	0	39
Cluster of Serum amyloid A-I protein	SAA1	14 kDa	10	23
Apolipoprotein A-II	APOA2	11 kDa	14	19
Afamin	AFAM	69 kDa	0	21
Clusterin	CLUS	52 kDa	0	23
Alpha-1-antitrypsin	A1AT	47 kDa	0	23
Haptoglobin	HPT	45 kDa	0	30
Apolipoprotein C-III	APOC3	11 kDa	9	13
Vitronectin	VTNC	54 kDa	0	19
Antithrombin-III	ANT3	53 kDa	0	14
Apolipoprotein E	APOE	36 kDa	3	14
Alpha-1β-glycoprotein	A1BG	54 kDa	0	13
Protein AMBP	AMBP	39 kDa	5	10
Serum paraxonase/arylesterase 1	PON1	40 kDa	10	0
Serum amyloid A-4 protein	SAA4	15 kDa	0	9
Ceruloplasmin	CERU	122 kDa	0	8
Kininogen-1	KNG1	72 kDa	0	8
Retinol-binding protein 4	RET4	23 kDa	0	9
Inter-alpha-trypsin inhibitor heavy chain H1	ITIH2	101 kDa	0	9
Alpha-2-HS-glycoprotein	FETUA	39 kDa	0	5
Apolipoprotein A-IV	APOA4	45 kDa	0	6
Apolipoprotein C-II	APOC2	11 kDa	0	2
Apolipoprotein D	APOD	21 kDa	0	4
Transthyretin	TTHY	16 kDa	0	3
Coagulation factor X	FA10	55 kDa	0	3

HTG: High Triglyceride; ALB: Albumin-enriched band; HDL: High-density lipoprotein; *TSC—Total Spectral Counts.

## Supplementary Materials

The following are available online at https://www.mdpi.com/2079-7737/8/3/53/s1, Table S1: Biochemical analysis of pooled human plasma samples; Figure S1: Dose response cholesterol exchange between VLDL and LDL; Figure S2: Calibration of LRA lipid particle PE and cholesterol fluorescence; Figure S3: Comparison of HDL *PE efflux and apoA-I content of pooled human plasma samples Figure S4: Transfer of LRA lipid particle fluorescent PE and cholesterol to human serum albumin.
