# Supplementary material for: ApoA-I-Mediated Lipoprotein Remodeling Monitored with a Fluorescent Phospholipid
Source: Biology (Basel). 2019 Jul 12;8(3):53. doi: 10.3390/biology8030053 (PMC6784057; doi:10.3390/biology8030053)
Supplement: Supplementary file 1 [file biology-08-00053-s001.pdf]

SupplementaryMaterials:

ApoA-I-Mediated lipoprotein Remodeling Monitored with a Fluorescent Phospholipid

Edward B. Neufeld, Masaki Sato, Scott M. Gordon, Vinay Durbhakula, Nicolas Francone, Angel Aponte, Gizem Yilmaz, Denis Sviridov, Maureen Sampson, Jingrong Tang, Milton Pryor and Alan T. Remaley

Table 1. Biochemical Analysis of Pooled Human Plasma Samples.

	LTG	HTG	LL	HH
**TC**	160	227	120	202
**HDL-C**	67	39	44	84
**TG**	38	413	115	61
**apoA-I**	154	139	138	193
**apoB**	70	116	56	80
**LDL-C**	86	106	54	105

All values are mg/dL; LTG: Low Triglyceride;HTG: High Triglyceride; LL: Low LDL; HH: High HDL.

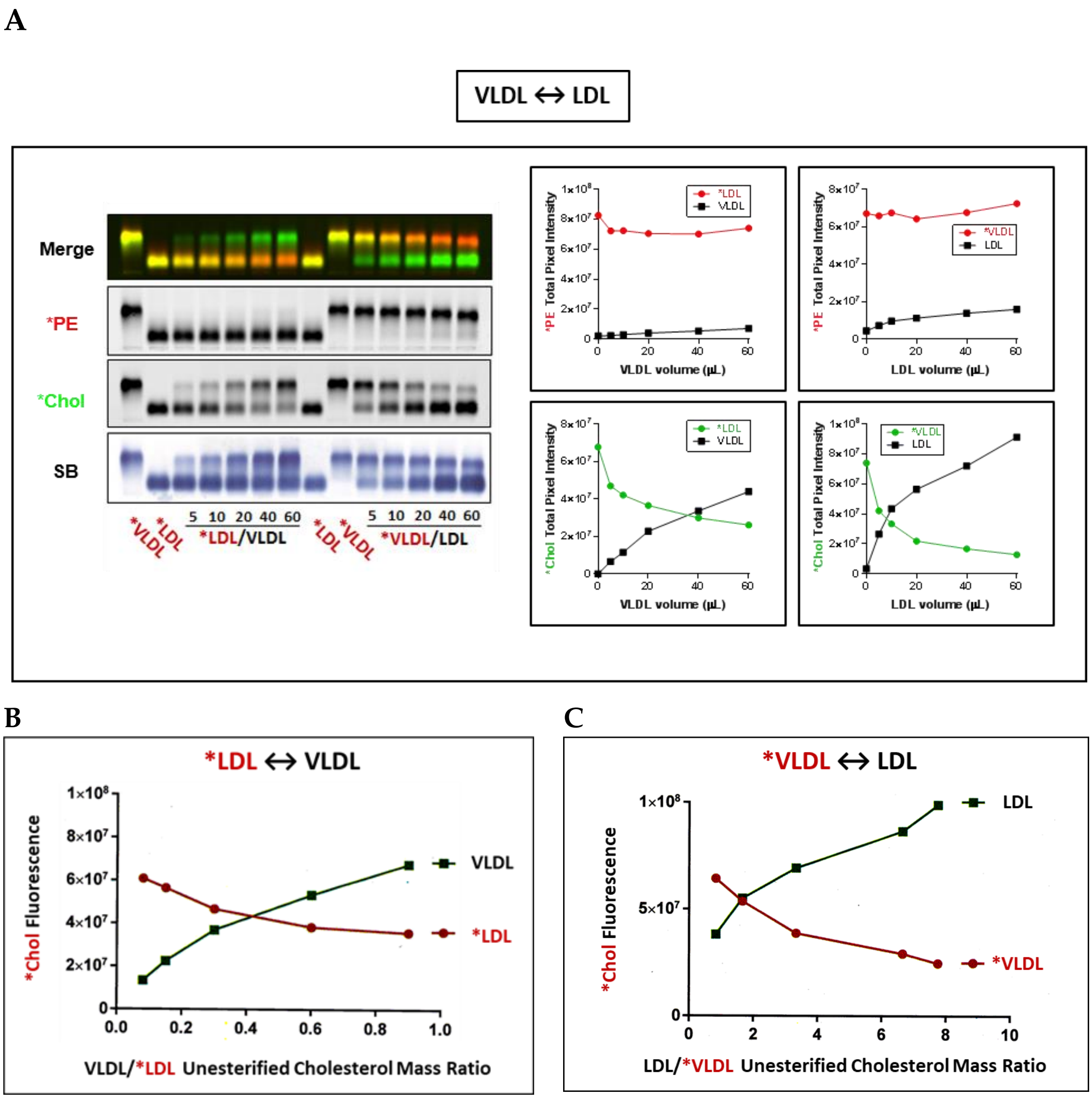

Figure S1. Dose response cholesterol exchange between VLDL and LDL. Agarose gel electrophoresis. Gel running times 90 min. (**A**) *VLDL and increasing volumes of unlabeled LDL (µL), and conversely, *LDL with increasing volumes of unlabeled VLDL (µL), were incubated for 4 h. Note that *PE remains associated with originally labeled lipoprotein. LDL appears to have a greater affinity for *Chol than VLDL, consistent with time course experiments shown in Figure 3C. Note that equal *Chol labeling of VLDL and LDL occurs with addition of 40 µL of unlabeled VLDL to *LDL and, 10 µL of unlabeled LDL to *VLDL, suggesting that LDL has a greater mass of unesterified cholesterol (UC). (**B**,**C**) We measured the UC content of the LDL and VLDL used in these experiments, and found that the apparent increased affinity of LDL for *Chol seen in (**A**) is simply due to the larger pool size of UC in the volumes of LDL used.

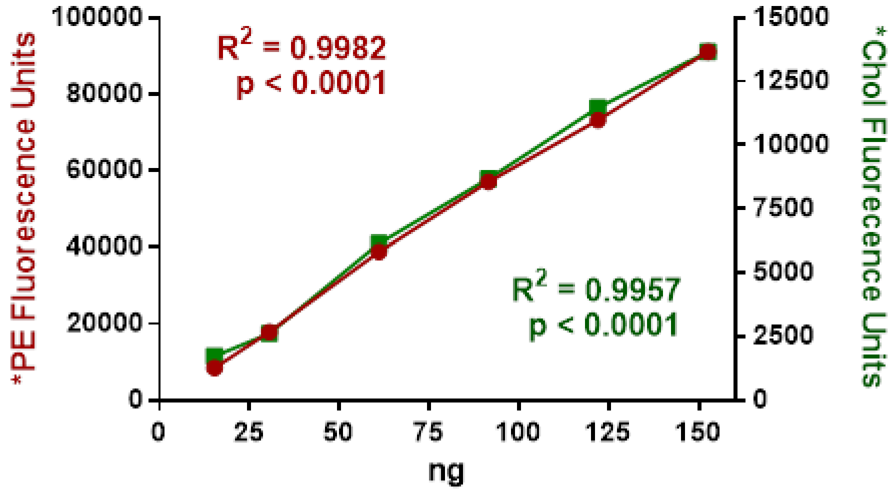

Figure S2. Calibration of LRA lipid particle PE and cholesterol fluorescence. Volumes of fluorescent lipid-tagged LRA lipid particles corresponding to the lipid masses shown were extracted with TX-100 detergent and PE and cholesterol fluorescence were measured as described in "Materials and Methods."

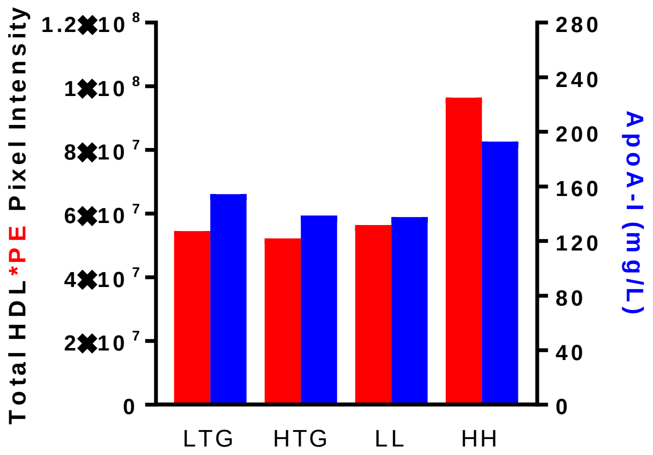

Figure S3. Comparison of HDL *PE efflux and apoA-I content of pooled human plasma samples. *PE HDL total pixel intensity (*red*) on agarose gels (Figure 5A) was measured using ImageQuant 5.1 software. ApoA-I values (*blue*) are those shown in Supplemental Table 1. Note that *PE HDL efflux appears to correlate with plasma apoA-I content.

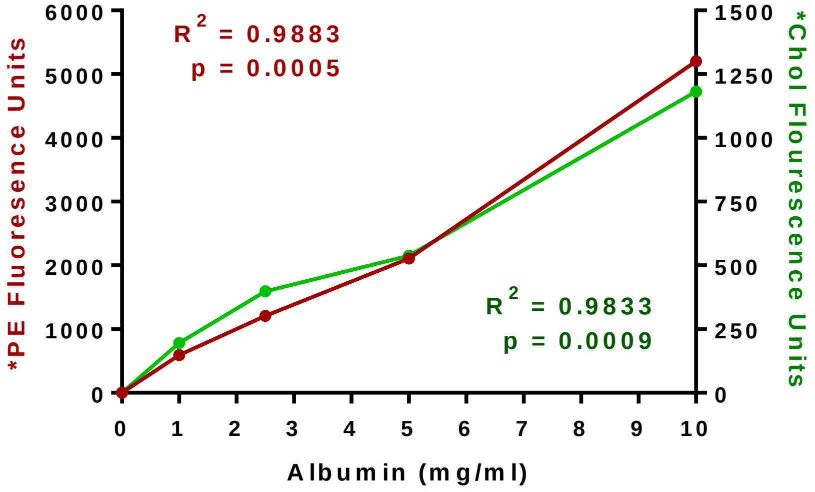

Figure S4. Transfer of LRA lipid particle fluorescent PE and cholesterol to human serum albumin. Human serum albumin was diluted with PBS to the concentrations shown and incubated with 60 mg LRA lipid particles labeled with fluorescent PE and cholesterol. Supernatants were extracted with 1% TX-100 detergent and PE and cholesterol fluorescence measured as described in "Materials and Methods."
